# Decoding high-order clinical correlations: a knowledge-driven large language model framework for specialized medical decision-making

**DOI:** 10.3389/frai.2026.1772518

**Published:** 2026-08-21

**Authors:** Qian Li, Yongxin Li, Chao Ye, Kaihua Zhang, Jing Bai, Xinbo Liu, Zhantao Li, Xuanyu Meng, Xianjun Min, Jin Guo

**Affiliations:** 1 Department of Thoracic Surgery, Beijing Genertec Aerospace Hospital, Beijing, China; 2 Department of Thoracic Surgery, Fourth Hospital of Hebei Medical University, Shijiazhuang, China; 3 School of Automation and Intelligence, Beijing Jiaotong University, Beijing, China; 4 School of Pharmacy, Hebei Medical University, Shijiazhuang, China; 5 Department of Pharmacy, Fourth Hospital of Hebei Medical University, Shijiazhuang, China; 6 Strategy Planning & Consulting Department, Genertec Digital Technology Co., Ltd., Beijing, China; 7 School of Medical Technology, Beijing Institute of Technology, Beijing, China

**Keywords:** domain-specific LLM, exam-style clinical reasoning, high-order clinical correlations, knowledge-augmented reasoning, medical NLP, thoracic surgery

## Abstract

**Background:**

Specialized thoracic-surgery questions require the integration of multi-factor clinical relationships within text, yet general-purpose large language models (LLMs) may underperform on such exam-style benchmarks.

**Methods:**

We constructed a DK-LLM agent by embedding curated medical textbook knowledge into a LangChain-based framework to support domain-specific reasoning. The model was tested on a 56-item thoracic-surgery examination question set in a restricted text-only setting without internet browsing or external tools and was compared with generic LLM baselines, three thoracic surgeons, and three non-expert engineers. Examination score and error patterns were assessed.

**Results:**

The knowledge-augmented DK-LLM configuration showed an 11.8-point examination-score advantage over the version without the local knowledge base. Commercial LLM-based agents outperformed the open-source baselines and non-expert participants on this question set, whereas experienced thoracic surgeons achieved the highest scores overall; in the ablation analysis, removing the local knowledge base reduced the examination score by 11.8 percentage points.

**Conclusion:**

Embedding domain-specific knowledge into LLMs may improve performance on specialized exam-style thoracic-surgery questions on this text-only benchmark. However, the present 56-item evaluation does not establish clinical equivalence, diagnostic accuracy in practice, multimodal competence, or readiness for real-world clinical decision support.

## Background

In recent years, the rapid accumulation of large-scale, multi-modal healthcare data has opened unprecedented opportunities in medical research. However, the complexity and heterogeneity of such data present significant analytical challenges. Diseases and their progression often depend on a multi-factorial interplay of genomic, proteomic, imaging, and clinical variables, and traditional analytical methods that focus on pairwise or low-order associations may fail to capture these intricate patterns. Consequently, there is a growing demand for high-order correlation mining techniques—such as hypergraph-based modeling, high-dimensional tensor decomposition, and multi-layer network analysis—that can more comprehensively characterize disease mechanisms, predict outcomes, and guide personalized interventions (Yang et al., 2022; Sun et al., 2022).

High-order correlation mining seeks to uncover complex, multi-way interactions hidden in high-dimensional and heterogeneous datasets. Recent studies have explored hypergraph structures that incorporate diverse data types (e.g., gene expression, metabolites, protein interactions, clinical phenotypes, and imaging features) into a single unified framework, capturing patterns that go beyond what simple pairwise models can represent (Sun et al., 2022). These approaches have demonstrated potential in patient subgroup discovery, identification of key biomarkers, and the refinement of treatment strategies. Yet, practical adoption remains challenging due to issues like data sparsity, noise, missing values, interpretability, and computational complexity. In this manuscript, the term “high-order clinical correlations” is used operationally for layered relationships among symptoms, examination findings, diagnoses, and management options within text-based specialist questions, rather than for direct patient-level multimodal network inference.

In the context of evaluating Large Language Models (LLMs) on specialized exam-style clinical reasoning, we utilized the Chinese National Senior Health Professional Technical Qualification Examination—a rigorous and widely recognized assessment for healthcare professionals in China. This examination evaluates a comprehensive set of skills, including diagnostic reasoning, treatment planning, and patient management, across various medical specialties. The decision to select this specific examination was based on its relevance to thoracic surgery and the need to assess LLMs’ ability to handle complex, domain-specific tasks that mirror structured specialist assessment scenarios. Given its stringent nature and focus on clinical proficiency, the examination provides a robust benchmark for evaluating the performance of LLMs in specialized medical fields, ensuring meaningful and applicable results.

Alongside these advances, LLMs have shown promise in various medical applications, including administrative tasks, telemedicine, clinical decision support, text summarization, and medical education (Wei et al., 2022; Chase, 2022; Arora and Arora, 2023; Sarraju et al., 2023). Models such as GPT-4 and ChatGPT have been evaluated on standardized medical exams (e.g., USMLE), radiation oncology physics tests, and Japanese medical licensing exams, demonstrating their potential for medical reasoning tasks (Liu et al., 2021; Edlow and Pronovost, 2023; Holmes et al., 2023; Kung et al., 2023; Oren et al., 2022; Yu et al., 2022). Domain-specific LLMs like GatorTron (Yang et al., 2022) and Med-BERT (Liu et al., 2021) have improved medical information extraction and entity recognition. However, current LLMs still face limitations when tackling high-order data relationships:

(1) Lack of multi-modal integration: most LLM-based approaches focus on text. They struggle to incorporate genomic, imaging, and clinical metrics into a unified analytical framework.
(2) Deficiencies in high-order reasoning: although LLMs show remarkable capabilities in language understanding, they lack explicit modeling strategies for complex, multi-way interactions (e.g., hypergraph-based or tensorized structures).
(3) Interpretability and trustworthiness: while LLMs produce fluent outputs, the reasoning behind their decisions remains opaque. This “black box” nature limits the trust clinicians can place in LLM-driven medical recommendations, particularly in high-stakes scenarios involving intricate multi-factorial decisions.

A promising direction lies in blending domain knowledge bases and advanced computational frameworks with LLMs. By integrating curated clinical guidelines, medical textbooks, and expert knowledge through frameworks like LangChain, which is an open-source Python framework that modularizes prompt templates, multi-step chains, dynamic agents, and memory management. It can support retrieval-augmented generation to seamlessly integrate large language models with external tools and data sources for complex workflow automation (Chase, 2022). LLMs gain targeted information that can help them reason about complex multi-dimensional patterns (Arora and Arora, 2023). Recent research also explores coupling LLM reasoning with high-order structures—such as hypergraphs or higher-dimensional embeddings—enabling not just plausible textual explanations, but also the capacity to discover meaningful patterns in complex, multi-modal clinical datasets. This interdisciplinary approach can move beyond current LLM bottlenecks and deliver practical tools for precision medicine and complex clinical case management.

Building on these insights, the proposed Domain-Specific Knowledge-Driven LLM (DK-LLM) agent aims to integrate domain-specific medical knowledge and advanced LLM capabilities within a knowledge-augmented reasoning framework. Unlike closed-book LLMs, the proposed system leverages structured textual knowledge to provide task-relevant context for thoracic surgery questions. We highlight three key innovations:

(1) Knowledge-augmented reasoning: by incorporating retrieval from a curated local knowledge base, the DK-LLM can use domain-relevant contextual information that is unavailable to standard closed-book LLM approaches.
(2) Domain specialization: evaluating performance against a stringent thoracic surgery examination ensures that the DK-LLM’s capabilities are truly adapted to the needs of a specialized clinical domain.
(3) Traceability and clinical relevance: retrieved knowledge snippets may improve the traceability of answers, although explainability, calibration, and clinician trust were not formally evaluated in this study.

Through these efforts, the proposed DK-LLM strives to provide an extensible and transparent framework for specialized medical question answering. In the present study, the implementation was restricted to textual knowledge retrieval and text-based examination questions rather than direct multimodal clinical input. The purpose of this study was to evaluate performance on thoracic-surgery examination questions, not to claim direct clinical competence. We hypothesized that integrating specialized medical knowledge would improve examination performance relative to closed-book baselines while still requiring further validation in real-world clinical settings.

## Methods

### Materials

#### Knowledge base construction

In this study, we employed the LangChain framework to establish a specialized textbook-based knowledge database sourced from four textbooks published by the Shanghai Medical College of Fudan University. The corpus comprised the two-volume “Practical Internal Medicine” and the two-volume “Practical Surgery.” No journal articles were included in the knowledge base used in this study. These publications provide broad coverage of internal medicine and surgery from foundational theories to clinical applications. The total size of the PDF files was 3.56 GB.

The textbooks “Practical Internal Medicine” extensively detail the diagnostic principles and therapeutic methods for various subspecialties within the field of internal medicine, including areas such as cardiovascular diseases, respiratory system diseases, and digestive system disorders. The information encapsulated within these texts is critical for understanding pathophysiological processes and clinical presentations, and for devising appropriate treatment strategies. This foundation is essential for decision-making in internal medicine.

Similarly, “Practical Surgery” encompasses the fundamental techniques of surgery, as well as perioperative management and specific treatment approaches for common surgical conditions. These volumes provide a rich source of specialized knowledge, particularly in managing acute and chronic diseases, trauma care, and therapeutic interventions during recovery phases.

To rigorously evaluate the performance of the DK-LLM agent, thoracic surgery was selected as the testing domain for several compelling reasons. First, thoracic surgery is a highly multidisciplinary and detail-sensitive field, encompassing complex clinical tasks such as the diagnosis and management of thoracic malignancies, lung function assessment, and surgical planning. These tasks often necessitate high-order pattern recognition to integrate anatomical, pathological, and procedural information, making thoracic surgery an ideal domain for benchmarking domain-specific large language models. Second, thoracic surgery is characterized by its diversity and complexity, with diagnostic challenges that reflect the heterogeneity of clinical practice in this specialty. Finally, the choice of thoracic surgery aligns with the broader goal of advancing AI applications in high-stakes medical fields, where decision-making intricacies are critical and errors can have significant consequences. By demonstrating the efficacy of the DK-LLM agent in this context, this study lays the foundation for future AI-driven advancements in specialized medical fields.

By integrating the LangChain framework, we transformed these textual resources into a structured local knowledge base that could be retrieved by Large Language Models (LLMs). We refined the extraction and indexing procedures to improve the accuracy and accessibility of the retrieved content. The resulting resource was intended as a restricted local reference for this experiment rather than a comprehensive or continuously updated evidence base. Accordingly, the present study evaluates whether textbook-based retrieval can improve performance on a specialized examination benchmark.

#### Evaluation materials

For evaluation, a total of 56 questions were gathered from the Chinese National Senior Health Professional Technical Qualification Examination (Thoracic Surgery). These questions were carefully selected by experienced medical experts specializing in thoracic surgery. The set of questions comprises 30 single-choice, 20 multi-choice, and 6 case-analysis questions, aligning with the established curriculum for resident education in thoracic surgery as recommended by the National Health Commission of the People’s Republic of China. The case-analysis items partially simulate a consultation-examination-treatment reasoning process, but the full benchmark remains an exam-style question set rather than a real-world clinical dataset.

To perform a quantitative assessment of the capabilities of the proposed agent, we conducted a benchmark analysis using the collected thoracic surgery examination questions. This analysis specifically focused on commonly used LLM APIs available as of April 28, 2024. A restricted evaluation setting was used: no internet browsing or external tools were permitted. The DK-LLM configuration could retrieve information only from the locally constructed textbook-based knowledge base, whereas the closed-book baseline LLMs had no retrieval and the human participants completed the examination without external aids. As for the scoring, the total score of the test is 100 points, typically consisting of 30 single-choice questions, 20 multi-choice questions, and 6 case-analysis questions. Concretely, the detailed scoring rules for each type of questions are summarized as follows:

Single-choice questions: 1 point is awarded for a correct answer, while no points are awarded for an incorrect answer or for not selecting an option.
Multi-choice questions: 2 points are awarded for selecting all correct options, while no points are awarded for selecting too many, too few, incorrect options, or not selecting any options.
Case-analysis questions: the correct answer(s) may involve one or multiple options. Each correct option selected earns 1 point, while each incorrect option selected results in a deduction of 1 point. The deduction continues until the score for that question reaches 0.

According to the training mechanism and text understanding and generation capabilities of LLM-based agent, in order to generate answers that are more in line with the perspective of a thoracic surgeon, we adopted a strategy. Before testing, we set the role of “thoracic surgeon” for each agent. This setting helps guide the model to better simulate the professional knowledge and language style of a thoracic surgeon when answering relevant questions, thereby maximizing the professionalism and accuracy of the generated answers.

### Models

**Figure 1:**
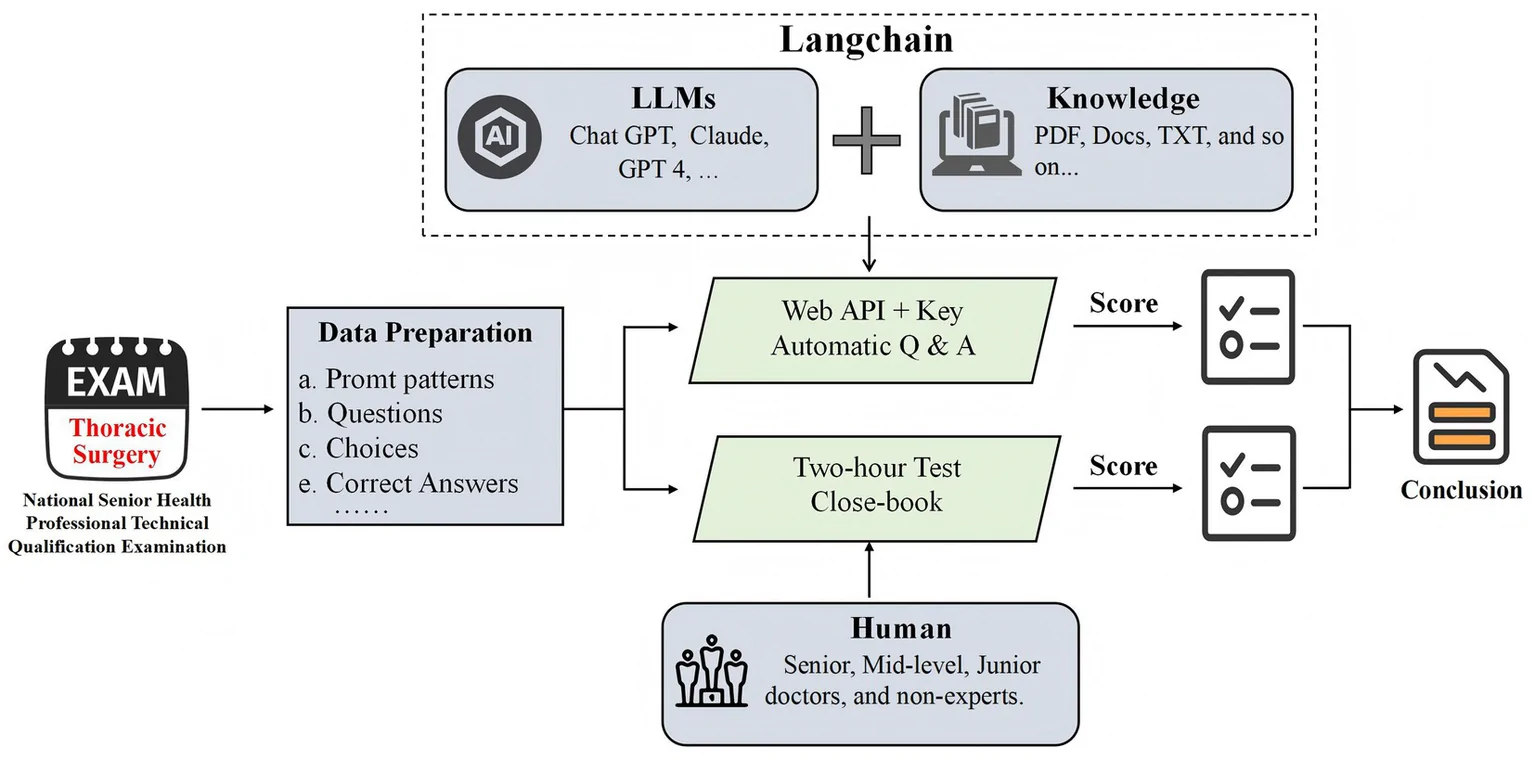
Revised workflow of the proposed DK-LLM agent under the restricted evaluation setting (local textbook retrieval only). The human benchmark comprised three thoracic surgeons and three non-expert engineers.

DK-LLM Agent development. In the development of the Domain-specific Knowledge-driven LLM (DK-LLM) agent (Figure 1), we started by developing a comprehensive local database that encapsulates specialized medical knowledge drawn from the four textbooks described above. This database was structured to support retrieval for a focused thoracic-surgery examination task rather than to serve as a comprehensive real-world clinical evidence system. The integration of this data into the LLM necessitated a detailed preprocessing workflow. Initially, we employed text segmentation algorithms designed to parse the medical texts into coherent, contextually meaningful segments. These algorithms are tailored to respect the semantic boundaries inherent in the medical literature, preserving the integrity of complex clinical concepts and terminologies.

Following segmentation, each text segment was subjected to a vectorization process. We utilized advanced natural language processing techniques to convert the textual data into numerical vectors, a prerequisite for compatibility with machine learning algorithms. Specifically, we applied embedding models capable of capturing the nuanced semantic relationships within the text, transforming them into high-dimensional vectors that retain the contextual essence of the original content. These vectors were systematically organized into a vector database, which forms the backbone of the proposed DK-LLM agent’s knowledge retrieval system. For efficient and contextually relevant information retrieval, the system harnesses cosine similarity measures. This approach facilitates the rapid identification of vectors that closely match the query inputs, enabling the DK-LLM to deliver precise and context-aware responses during clinical decision-making processes. For reproducibility, the released retrieval pipeline used a recursive character splitter with a chunk size of 1,000 characters and an overlap of 200 characters, with chunk length counted after whitespace removal.

To enhance the DK-LLM’s performance with domain-specific knowledge, we employed LangChain as the core framework for retrieval-augmented generation (RAG), facilitating seamless integration of a structured knowledge base with the LLM for real-time access to relevant textual clinical information during inference. LangChain indexed the knowledge base in FAISS using text-embedding-3-large embeddings; during inference, similarity search retrieved the top 3 most relevant chunks for each query, and these snippets were inserted into the prompt context. This RAG-based approach, powered by LangChain, augmented the DK-LLM’s reasoning on the examination benchmark; it did not involve direct processing of imaging files or other non-text modalities in the current study.

In the knowledge-base integration phase of this study, we constructed a comprehensive OCR and text-parsing pipeline in Python to extract high-quality, structured information from scanned medical materials and PDF documents. Specifically, we employed the pdf2image library to convert each PDF page into high-resolution images, followed by pytesseract to perform OCR, generating preliminary plain text. Subsequently, the extracted text was segmented, denoised, and subjected to clinically relevant named entity recognition (NER)—covering diseases, anatomical structures, medications, and diagnostic metrics—using Python-based natural language processing tools such as spaCy or scispaCy. We then utilized regular expressions and a rule-based engine to correct noisy characters and common OCR confusions, simultaneously establishing hierarchical mappings between sections and labels to ensure consistent indexing and retrieval. Finally, all preprocessed text, along with the corresponding entities and contextual information, was stored in a structured database (e.g., SQL or a document-oriented database), providing a queryable knowledge source to the LangChain framework for real-time access and integration by the LLM. This Python-driven, multi-stage technical pipeline produced a text-centric medical knowledge base; direct multimodal analysis was not performed in the present evaluation.

Further integrating the LangChain framework with LLM technology, we crafted a system that leverages structured knowledge to assist with specialized exam-style reasoning. This integration is pivotal for the DK-LLM agent, enabling it to interpret and respond effectively to complex thoracic-surgery questions. The methodology underwent iterative optimization in a controlled evaluation setting based on examination questions rather than prospective clinical workflows.

#### Baseline models

We evaluated the proposed framework against six baseline model settings: GPT-4, GPT-3.5-turbo, Claude, Med-PaLM, ChatGLM-6B (Du et al., 2021), and FastChat/Vicuna. The commercial models were accessed through provider-hosted interfaces available to the authors on April 28, 2024; for Med-PaLM, the exact provider-side checkpoint/version was not disclosed to the authors. All baseline models were evaluated in the same closed-book text-only setting using the zero-shot, one-shot, and five-shot prompts described below, and no retrieval, internet browsing, plugins, or external tools were enabled. The open-source baselines were transformer-based models available through their public repositories (Han et al., 2021), whereas low-level training details for the commercial baselines were not fully public.

In this study, we focus on using the LLM-based Agents to tackle Thoracic Surgery questions from the Chinese National Senior Health Professional Technical Qualification Examination. By assessing their performance in this specialized domain, we aim to validate their capabilities in clinical applications.

#### Prompting and output formatting

**Figure 2:**
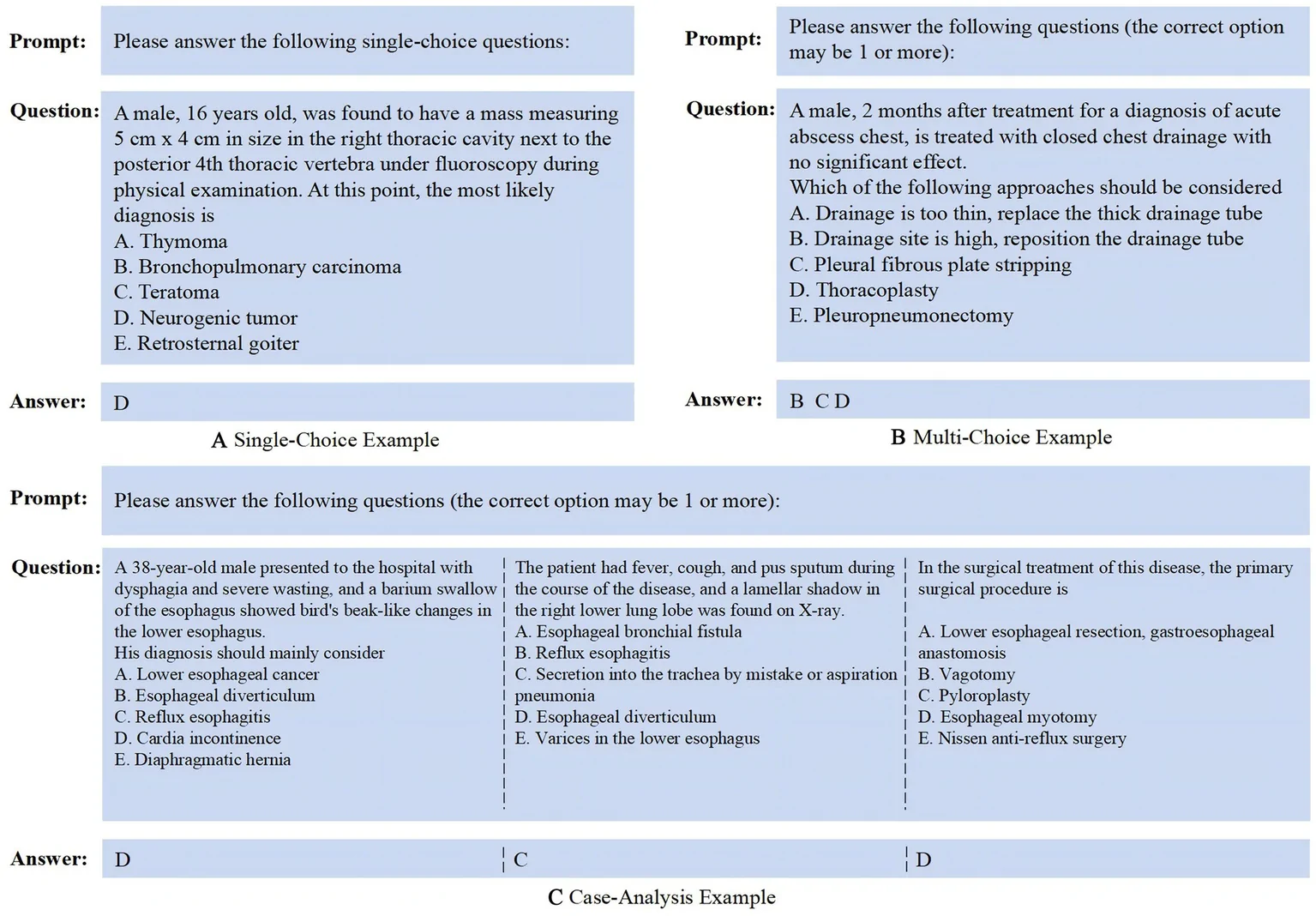
Our prompts for DK-LLM agents. English translations are provided here for the evaluation. Here randomly selects questions from each type of questions.

Prompts are crucial in guiding LLM agent responses. Table 1 shows the prompts used for each question type, and Figure 2 demonstrates their concise utilization in representative examples: single-choice, multi-choice, and case-analysis questions from the Thoracic Surgery examination. This illustrates the effective use of prompts in guiding Agent responses to specific question types.

**Table 1:** The LLM prompts used in each type of questions. ${Questions} denotes the examined question in each test.

Question type	Prompt
Single-choice	Please answer the following single-choice questions: ${Questions}
Multi-choice	Please answer the following questions (the correct option may be 1 or more): {Questions}
Case-analysis	Please answer the following questions (the correct option may be 1 or more): {Questions}

To simulate the role of a thoracic surgeon within the DK-LLM framework, we employed explicit role instructions and contextual cues to guide the model’s response style. For the present study, these prompts were designed for text-based examination questions rather than direct analysis of raw multimodal clinical inputs.

“You are a thoracic surgeon. Please answer the following thoracic-surgery examination question using the provided text and, when enabled, the retrieved local knowledge snippets. Base your response on standard clinical knowledge and select the most appropriate answer option(s).”

In addition to the general role prompt, task-oriented instructions were adapted to the single-choice, multi-choice, and case-analysis formats used in the examination.

The prompts did not include raw CT images, pathology slides, or laboratory time series as direct model inputs in this study.

Accordingly, the current evaluation should be interpreted as a text-based benchmark rather than a true multimodal assessment.

These prompt templates were iteratively refined through pilot testing to ensure alignment with examination-style clinical reasoning processes. The inclusion of these predefined prompts maintained focus on thoracic surgery-specific considerations, but it does not constitute a formal evaluation of interpretability or clinician trust.

The examination questions were originally administered in Chinese. For manuscript presentation, the example prompts shown in Figure 2 were translated into English by two bilingual authors and checked by a thoracic surgeon to preserve the intended clinical meaning.

No model retraining or in-study teaching of translation rules was performed. Unless explicitly stated otherwise, all benchmark scores reported in this manuscript were obtained from the original Chinese-language questions; the translated English text is shown only for reader accessibility and is not analyzed as a separate primary result set.

In the study, we also analyzed the role of additional contextual information for agent predictions. We found that contextual information providing prerequisites is usually beneficial to the agent’s predictions, for example, in the title “Male, 26 years old, stab wound to the chest for 20 min, emergency admission to the operating room, complained of thirst, patient irritability, physical examination: blood pressure 92/71 mmHg, heart rate 116 beats/min, weak heart sounds, no murmur heard in the heart, clear breath sounds in both lungs, clear percussion, a 2-cm-long incision in the lower sternum near the glabella, no active bleeding, ultrasound examination of fluid in the pericardial cavity blood pressure dropped to 71/50 mmHg, possible diagnosis,” the title provides the patient’s details, and the LLM agent relies on this information to conclude that the most likely diagnosis is pericardial effusion. The patient had a history of a stab wound to the chest, and an ultrasound showed fluid in the pericardial cavity. Other options, such as acute pericarditis, chronic restrictive pericarditis, and pericardial effusion, were inconsistent with the patient’s clinical presentation and findings. However, in some cases, additional contextual information may not contribute to the agent’s predictions, such as in the question “Which of the following is an indication for closed pleural cavity drainage in the case of thoracic injury?,” where the information provided about the thoracic injury is additional contextual information, and the LLM agent accurately captures the question’s key point, “closed pleural cavity drainage,” and analyzes it to give the correct answer. However, if redundant contextual information is provided in the question, it may sometimes mislead the agent’s prediction. For example, in the question “Male, 50 years old, swallowing foreign body for 1 month, previous history of hepatitis B for 20 years, further examination should be considered first,” the agent’s initial prediction only caught the supplementary information “previous history of hepatitis B for 20 years” and made a wrong prediction. In this case, we could have focused on the patient’s existing information, pointed out the primary consideration in the question, and asked the LLM agent to make a prediction, which was correct.

We evaluated the effectiveness of direct prompts and example-based prompts for language model testing. We conducted experiments with zero, one, and five examples. We also tested prompts requesting intermediate rationale to examine whether more structured responses improved performance. Specific example prompts can be seen in Figure 2.

During the evaluation phase, we meticulously configured key parameters to optimize the balance between response consistency and contextual relevance. The temperature parameter, which regulates the randomness of the model’s outputs, was set to 0 for the optimal experiments. This configuration ensured that the generated responses were highly deterministic and aligned with the specific clinical scenarios under evaluation. Higher temperatures, tested in preliminary experiments (e.g., 0.7), resulted in responses with increased variability but diminished alignment with clinical expectations, whereas lower temperatures (e.g., 0.3) offered limited diversity without significant gains in contextual accuracy.

Furthermore, we explored prompting strategies that requested intermediate explanatory steps to assess whether they could enhance examination performance. Consistent with prior findings, including studies on law school exam evaluations, these methods did not yield substantial improvements (Choi et al., 2021).

The experiment also revealed some questions that were not adequately supported, resulting in LLM agents providing incorrect answers. For instance, the question “The early symptoms of esophageal cancer are” lacked any indications or clues, leading agents to rely on their own understanding, which may have resulted in inaccurate responses.

### Evaluations

To evaluate model performance, we automatically scored responses according to the official examination rubric. Single-choice and multi-choice items were scored dichotomously according to the predefined answer key, whereas case-analysis items followed the partial-credit rules described above. This differs from open-ended generation tasks that often require human evaluations or more advanced metrics16. Test scores were determined as per Section 5.1. We also compared agents’ scores with human scores, involving two groups: thoracic surgeons and non-experts. The surgeons had substantial thoracic surgery experience, while the non-experts held advanced degrees in technical fields but lacked thoracic surgery knowledge.

Each human participant completed a two-hour closed-book test without external aids. In comparing agent performance with expert benchmark scores, we calculated mean scores and summarized observed score variability across repeated LLM runs. For the LLM agents, each question was repeated three times under the same prompt setting to estimate run-to-run variability in total score. Formal answer-confidence, calibration, and correlation analyses were not performed in the present study.

### Statistical analysis section

Data were analyzed using SPSS version 26. Because all agents were evaluated on the same 56 questions, the resulting observations were not fully independent. Accordingly, score comparisons are presented primarily as descriptive statistics, and the reported t tests should be interpreted as exploratory comparisons of aggregate total scores rather than formal equivalence testing. No mixed-effects analysis or calibration assessment was performed in the current study.

## Results

### Overall evaluation

**Figure 3:**
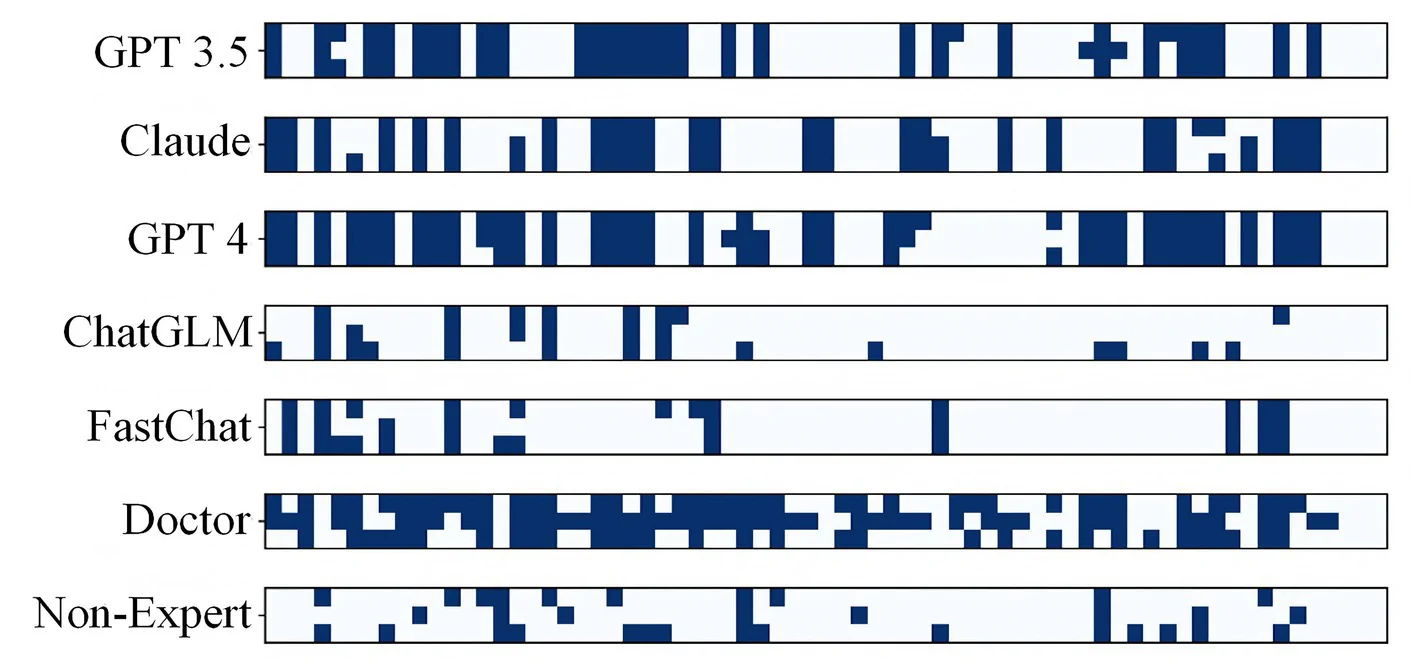
Raw marks for each test where the rows are separate tests and the columns are the test questions. Dark shaded squares represent correct answers. According to the raw scores, GPT-4 outperforms the other LLMs and shows the smallest gap relative to the doctor group.

The evaluation involved three experienced thoracic surgeons, each with at least 10 years of clinical experience in thoracic surgery. Their performance was compared to that of LLM agents. The top-performing model setting, five-shot GPT-4, scored 48.67 out of 100, which was 10.89 points lower than the mean surgeon score. Exploratory comparisons yielded *p* > 0.05 for GPT 3.5 and GPT 4 versus the surgeon group (Table 2 and Figure 3), but this non-significant result should be interpreted cautiously because the 56-item benchmark and the human sample were small and do not establish equivalence.

**Table 2:** Test scores on national senior health professional technical qualification examination (thoracic surgery).

Agent	Source	Zero-shot	One-shot	Five-shot
GPT 3.5	Commercial	38.00	41.33	43.67
Claude	Commercial	37.67	29.67	41.67
GPT 4	Commercial	**48.0**	**46.0**	**48.67**
Med-PaLM	Commercial	43.6	42.5	45.2
ChatGLM	Open source	10.0	5.67	6.33
FastChat	Open source	16.33	25.0	12.0
Thoracic surgeon 1	Human	58.67	NA	NA
Thoracic surgeon 2	Human	60.67	NA	NA
Thoracic surgeon 3	Human	59.33	NA	NA
Non-expert 1	Human	23.33	NA	NA
Non-expert 2	Human	21.00	NA	NA
Non-expert 3	Human	19.67	NA	NA

Human participants completed a single closed-book test; therefore, the prompt-condition columns are not applicable to those rows.

The bold and underlined values are the best and second-best model performance, respectively. Zero-shot, one-shot, and five-shot refer to LLM prompting conditions. For human participants, a single closed-book score is reported and the one-shot/five-shot columns are not applicable.

Given the shared 56-question set and the limited number of human participants, we revised the statistical interpretation to be descriptive and exploratory rather than definitive. Commercial LLMs generally scored higher than the open-source models and the non-expert group, whereas the thoracic surgeons achieved the highest overall scores. Exploratory t tests comparing GPT 3.5 or GPT 4 with the surgeon group yielded *p* > 0.05, but these findings do not demonstrate expert equivalence. Overall, the results suggest that domain-specific knowledge and stronger commercial LLMs can improve performance on specialized exam-style questions, while additional validation on larger and clinically grounded datasets remains necessary.

### Comparison between LLMs and expert benchmark scores

As shown in Table 2, the benchmark comparison involved three thoracic surgeons and three non-expert engineers, whose anonymized assessments were used solely for performance reference. While the GPT-4-based agent outperformed the other LLMs, it remained 10.89 points below the mean surgeon score. The non-expert humans scored 19.67 on average, lower than all commercial LLM-based agents, suggesting that these models can answer some thoracic-surgery questions better than non-experts but still do not match experienced surgeons on this benchmark.

### Observed score variability across repeated runs

**Figure 4:**
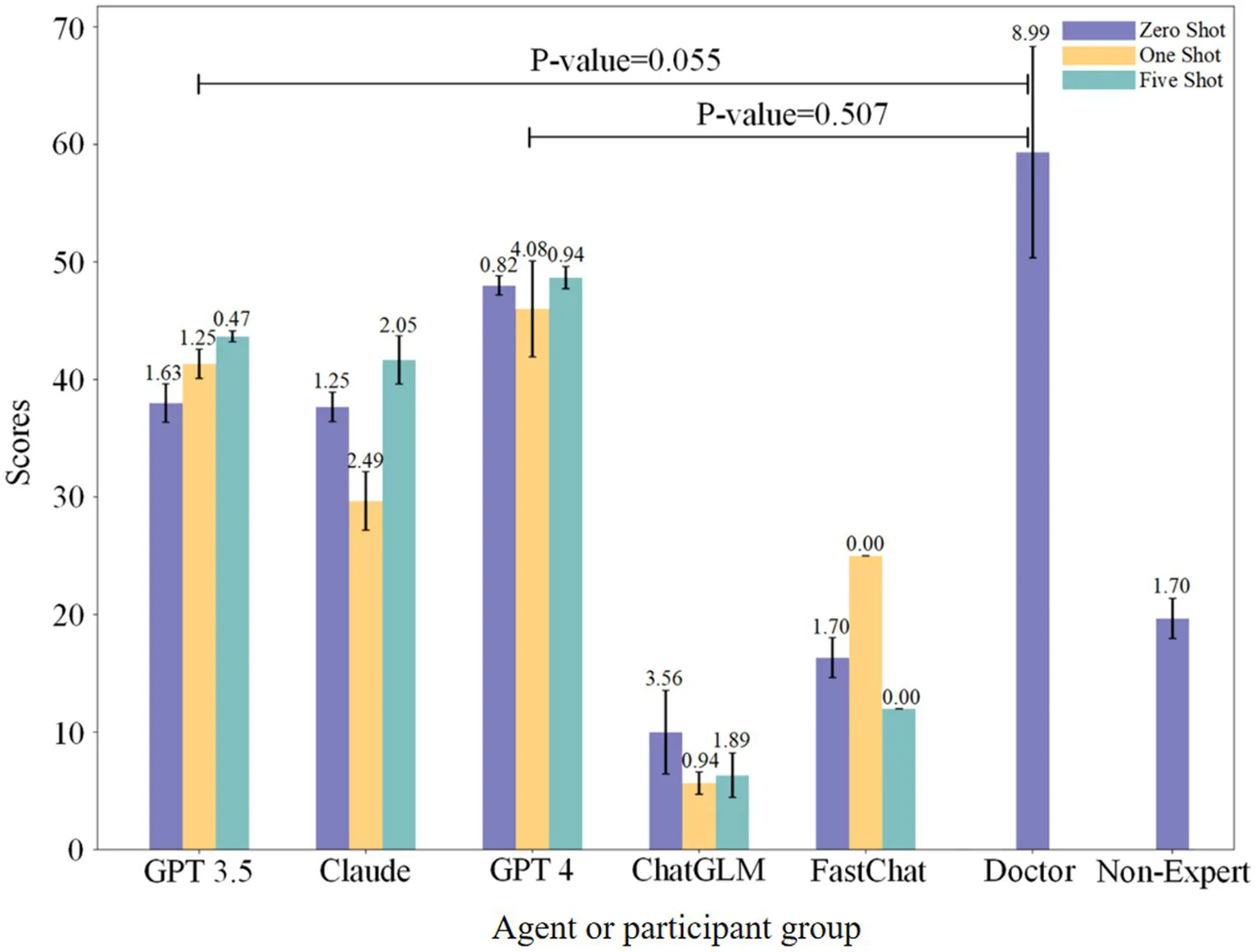
Test scores of LLM agents under zero-shot, one-shot, and five-shot prompting, together with the mean ± SD of the human single-test scores. The human bars do not represent prompting conditions; they summarize the three thoracic surgeons and the three non-expert participants, respectively. The *p* values from the exploratory *t* tests comparing GPT 3.5 with doctors and GPT 4 with doctors are shown above the bars.

Figure 4 presents the test scores of LLM agents together with the observed variability across repeated runs and the distribution of the single-test human scores. While doctors obtained higher scores overall, LLMs generated responses within seconds. Response speed may be an operational advantage, but in the present study it did not offset the score gap between the top LLM configurations and the surgeon group.

### Impact of giving examples

In the experiments, we employed Zero-Shot, One-Shot, and Few-Shot prompt settings to evaluate the DK-LLM’s performance. These settings vary in the number of examples provided to the model, influencing its ability to generate relevant responses. Zero-Shot involves no examples, where the model generates a response based purely on its pre-trained knowledge; One-Shot provides a single example to guide the model’s response, establishing basic context; Few-Shot (e.g., five-shot) offers multiple examples, enabling the model to better capture complex patterns and improve performance on more intricate tasks.

These prompt types are widely used in LLM evaluations to assess the model’s generalization and contextual adaptation capabilities. In the study, they allowed us to examine how varying levels of guidance impacted the DK-LLM and benchmark models’ performance.

Figure 4 shows each agent’s test scores under these settings. More examples improved some LLM agents like GPT 3.5, Claude, and GPT-4, but not all. For instance, GPT 3.5 scored higher in one-shot and five-shot than in zero-shot. The best-performing agent, GPT-4-based one, scored lowest in one-shot but highest in five-shot, outperforming GPT-3.5’s five-shot score even in its zero-shot setting-based agents.

### Performance on different question types

**Figure 5:**
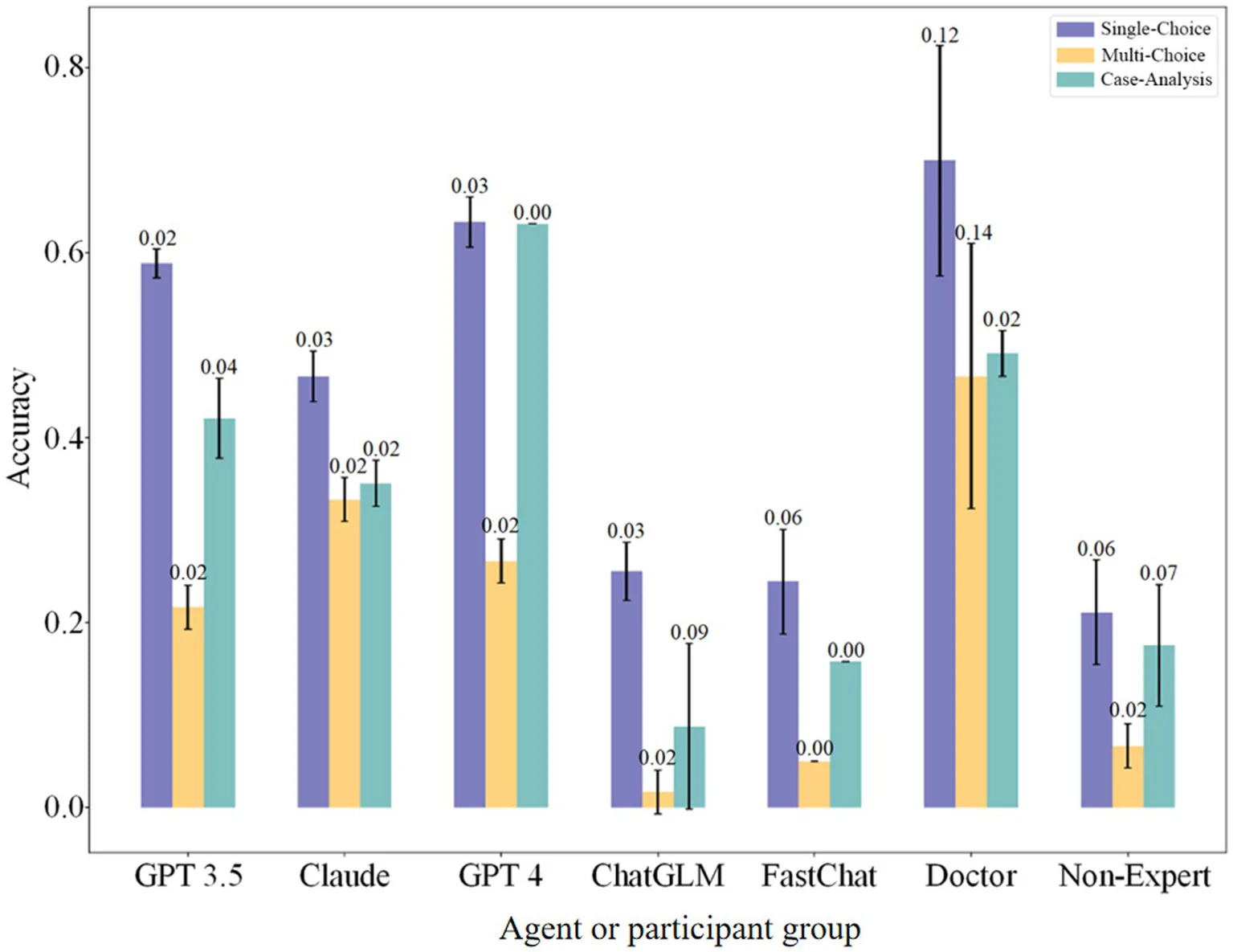
The detailed results of each type of questions. The error bar is indicated with their values. The visualization shows agent’s proficiency across single-choice, multi-choice, and case analysis questions in a zero-shot setting.

Figure 5 shows agent’s proficiency across single-choice, multi-choice, and case analysis questions in a zero-shot setting. Both agents and humans performed better on single-choice questions and struggled with multi-choice questions. Scores were higher for case-analysis questions because related sub-questions provided additional local context within the same item. The GPT-4-based agent, the top-performing LLM configuration, achieved comparatively strong results on single-choice and case-analysis questions, but these findings still reflect exam-style reasoning rather than validated clinical competence.

We performed a comparative analysis between the DK-LLM and Med-PaLM to evaluate domain-specific performance on thoracic-surgery examination tasks. As presented in Table 2, DK-LLM demonstrated an average improvement of 3.79% in examination score compared to Med-PaLM across zero-shot, one-shot, and five-shot scenarios. Med-PaLM nevertheless performed strongly relative to several other baselines. The observed advantage of DK-LLM may reflect the contribution of the structured domain-specific knowledge base, although the small benchmark prevents strong conclusions about general clinical performance.

To further elucidate the contribution of the knowledge base to DK-LLM’s efficacy, we conducted an ablation study. The ablation used the same GPT-4-based five-shot DK-LLM configuration as the top-performing setting in Table 2 (temperature = 0), with local textbook retrieval toggled on or off while all other settings were held constant. In this experiment, the model operated without the external knowledge base, relying solely on its pretrained parameters and intrinsic knowledge. The results, summarized in Table 3, revealed a marked decline in performance metrics. Specifically, the examination score dropped by 11.8 percentage points, emphasizing the critical role of the structured knowledge base in improving performance on this benchmark. Because “high-order correlation recall” was not formally predefined or annotated in the current study, that metric has been removed from the revised interpretation.

**Table 3:** Performance comparison of DK-LLM with and without knowledge base.

Metric	With knowledge base	Without knowledge base	Difference
Examination score (%)	48.67	36.87	−11.8

The ablation used the GPT-4-based five-shot configuration (temperature = 0), with all non-retrieval settings held constant.

Collectively, these findings underscore the contribution of the local knowledge base to DK-LLM performance on this specialized examination benchmark. However, the present results should be interpreted as evidence of improved exam-style question answering rather than proof of real-world clinical reasoning, multimodal competence, or interpretability.

### Clinical decision support

In the present study, the evaluation set consisted of examination questions rather than prospectively collected clinical cases. The six case-analysis items partially simulated consultation-examination-treatment reasoning pathways, so we interpret this section as an analysis of exam-style clinical reasoning proxies rather than validated clinical decision support.

A representative failure mode was observed in the question concerning a 50-year-old man with a foreign-body ingestion history and chronic hepatitis B. The model over-weighted the hepatitis history and incorrectly prioritized esophageal cancer, whereas the expected answer focused on the most appropriate next examination under the exam rubric. This example illustrates how irrelevant contextual details can misdirect model reasoning even in a text-only question setting.

These examples illustrate both the potential and the limitations of DK-LLM on exam-style reasoning tasks. They should not be interpreted as validated real-time clinical decision support. No prospective clinical deployment, formal expert-consensus study, or uncertainty-calibration analysis was performed in the current work.

## Discussion

The proposed DK-LLM agent exemplifies how integrating domain-specific medical knowledge with LLM-based reasoning frameworks can improve performance on specialized medical question-answering tasks. By linking curated domain knowledge with an advanced LLM, we address some limitations of standard closed-book LLMs while keeping the claims within the scope of a text-based thoracic-surgery benchmark.

This study introduces the Domain-Specific Knowledge-Driven LLM (DK-LLM) agent, which bridges the gap between generic language models and the needs of a specialized examination task. By embedding authoritative medical knowledge within the LangChain framework, the DK-LLM adapts its responses to thoracic-surgery questions and improves examination performance relative to non-knowledge-augmented settings.

The core innovation lies in the integration of a structured local knowledge base that provides domain-relevant textual context during inference. In the present study, this knowledge base was derived from authoritative medical texts and converted into retrievable vectors for a text-based benchmark. The current implementation does not directly analyze imaging, laboratory data, or other non-text modalities.

We rigorously benchmarked the DK-LLM using the Chinese National Senior Health Professional Technical Qualification Examination in thoracic surgery as a constrained, exam-style evaluation. This benchmark tests the agent’s ability to process layered textual questions, but it should not be interpreted as a direct substitute for real-world patient care or prospective clinical validation.

While GPT-4 and similar commercial models outperformed open-source models, they still lag behind seasoned clinicians. High-dimensional, specialized exams—like the thoracic surgery evaluation used here—pose greater challenges than broad-based medical tests (e.g., USMLE)10. The non-significant exploratory comparisons with surgeons should therefore not be interpreted as evidence of expert equivalence. This underscores the need for approaches that combine knowledge-driven frameworks with richer evaluation on larger, clinically grounded datasets.

In this study, we focused on applying the Domain-Specific Knowledge-Driven LLM (DK-LLM) agent within thoracic surgery, where the integration of domain-specific knowledge improved examination performance. However, the current study remained limited to a text-based benchmark and did not evaluate direct multimodal reasoning or clinical workflow integration. The modular design of the knowledge base and the adaptability of the LangChain framework may nevertheless allow extension to other medical disciplines in future work. Recent non-English board-examination studies also suggest that augmentation, multilinguality, and calibration should be treated as distinct methodological issues rather than as a single performance dimension.

Recent non-English examination studies provide useful methodological context. Kasai et al. evaluated GPT systems on the Japanese Medical Licensing Examinations, whereas Madrid et al. separately examined plugin augmentation, pretranslated inputs, and a formal uncertainty metric (“confidence accuracy”) on the German Medical Board Examination. Relative to these studies, the present work isolates a local textbook-RAG intervention in Chinese and therefore informs retrieval augmentation more directly than multilingual transfer or formal calibration. Future directions will also include expanding the DK-LLM framework to additional disease areas, such as cardiology, oncology, and neurology, while preserving domain specificity in each deployment setting (Kasai et al., 2023; Madrid et al., 2025).

The knowledge base was developed exclusively in Chinese, which confined the model’s construction and primary evaluation to this language. This constraint highlights the challenges involved in building a robust multilingual knowledge base and reinforces the need to evaluate language transfer separately from retrieval augmentation.

Future work will focus on expanding the DK-LLM framework to multiple languages while preserving domain specificity. Multilingual generalization was not formally benchmarked in the present study; the English text shown in Figure 2 was prepared for reader accessibility rather than analyzed as a separate validated result set. Future multilingual evaluations should therefore be paired with explicit uncertainty or calibration metrics.

Future work will evaluate the DK-LLM agent on prospectively curated thoracic-surgery case sets with structured annotations, and may incorporate imaging, laboratory findings, and other multimodal data when such datasets are available.

Despite these measures, several limitations should be emphasized. The benchmark included only 56 questions and six human participants, which limits statistical power and generalizability. Examination performance is not equivalent to real clinical competence, uncertainty management, or patient-safety performance. The current DK-LLM is text-based and does not directly analyze imaging, pathology, waveform, or other multimodal inputs. Because the knowledge base was derived from textbooks that overlap with the examination domain, we cannot exclude content overlap or memorization effects. In addition, explainability, clinician trust, answer confidence, and calibration were not formally evaluated.

Emerging reasoning-oriented models may further enhance adaptability and understanding of complex clinical contexts. However, challenges remain: maintaining updated domain knowledge, establishing calibration and interpretability, and achieving efficient deployment in real clinical workflows. These next-generation models must be paired with curated knowledge bases, prospective validation, and regulatory oversight to ensure trustworthiness and patient safety.

As these technologies mature, integrating LLMs with domain-specific knowledge, multi-modal medical data (genomics, imaging), and evolving clinical guidelines can yield context-aware, explainable, and timely decision support. Although not yet a substitute for experienced clinicians, refined knowledge-driven frameworks like DK-LLM hold the promise of mitigating expertise shortages, enhancing diagnostic precision, and ultimately improving patient outcomes.

## Conclusion

The Domain-Specific Knowledge-Driven LLM (DK-LLM) agent marks a step toward applying AI to specialized clinical question answering. By fusing deep domain knowledge with LLM reasoning capabilities, we aim to narrow the gap between general-purpose NLP models and the more specialized reasoning required by thoracic-surgery examination tasks.

Evaluations against specialized thoracic surgery questions suggest that the DK-LLM agent can improve performance relative to non-knowledge-augmented settings on this benchmark. Future work should refine the algorithms, expand dataset size, improve calibration and interpretability assessment, and ensure rigorous ethical and data-governance procedures. While the current results do not establish expert equivalence or clinical readiness, they support continued investigation of knowledge-driven medical AI tools for specialized domains.

## Data Availability

The datasets presented in this study can be found in online repositories. The names of the repository/repositories and accession number(s) can be found at https://github.com/qianli-ai/KnowledgeDrivenLLM.

## References

[ref1] Arora A. Arora A. (2023). The promise of large language models in health care. Lancet 401:641. doi: 10.1016/S0140-6736(23)00216-7, 36841609

[ref2] Chase H. LangChain: A Framework for Developing Applications Powered by Language Models. GitHub. (2022). Available online at: https://github.com/hwchase17/langchain (Accessed June 15, 2024).

[ref3] Choi J. H. Hickman K. E. Monahan A. B. Schwarcz D. (2021). ChatGPT goes to law school. J. Legal Educ. 71:387.

[ref4] Du Z. Qian Y. Liu X. Ding M. Qiu J. Yang Z. . (2022). Glm: general language model pretraining with autoregressive blank infilling. In Proceedings of the 60th Annual Meeting of the Association for Computational Linguistics (Volume 1: Long Papers), 320–335. doi: 10.1109/GLM2021

[ref5] Edlow J. A. Pronovost P. J. (2023). Misdiagnosis in the emergency department: time for a system solution. JAMA 329, 631–632. doi: 10.1001/jama.2023.0577, 36705932

[ref6] Han K. Xiao A. Wu E. Guo J. Xu C. Wang Y. (2021). Transformer in transformer. Adv. Neural Inf. Process. Syst. 34, 15908–15919. doi: 10.5555/3540261.3541478

[ref7] Holmes J. Liu Z. Zhang L. Ding Y. Sio T. T. McGee L. A. . (2023). Evaluating large language models on a highly-specialized topic, radiation oncology physics. Front. Oncol. 13:1219326. doi: 10.3389/fonc.2023.1219326, 37529688 PMC10388568

[ref8] Kasai J. Kasai Y. Sakaguchi K. Yamada Y. Radev D. (2023). Evaluating GPT-4 and ChatGPT on Japanese medical licensing examinations. arXiv. [Preprint] doi: 10.48550/arXiv.230318027

[ref9] Kung T. H. Cheatham M. Medenilla A. Sillos C. de Leon L. Elepaño C. . (2023). Performance of ChatGPT on USMLE: potential for AI-assisted medical education using large language models. PLoS Digit. Health 2:e0000198. doi: 10.1371/journal.pdig.0000198, 36812645 PMC9931230

[ref10] Liu N. Hu Q. Xu H. Xu X. Chen M. (2021). Med-BERT: a pretraining framework for medical records named entity recognition. IEEE Trans. Ind. Inform. 18, 5600–5608. doi: 10.1109/TII.2021.3131180

[ref11] Madrid J. Diehl P. Selig M. Rolauffs B. Hans F. P. Busch H. J. . (2025). Performance of plug-in augmented ChatGPT and its ability to quantify uncertainty: simulation study on the German medical board examination. JMIR Med Educ. 11:e58375. doi: 10.2196/58375, 40116759 PMC11951815

[ref12] Oren O. Blankstein R. Bhatt D. L. (2022). Addressing imaging pitfalls to reduce cardiovascular disease misdiagnosis in patients with breast cancer following reconstruction. JAMA Cardiol. 7, 123–125. doi: 10.1001/jamacardio.2021.4564, 34730778

[ref13] Sarraju A. Bruemmer D. Van Iterson E. Cho L. Rodriguez F. Laffin L. (2023). Appropriateness of cardiovascular disease prevention recommendations obtained from a popular online chat-based artificial intelligence model. JAMA 329, 842–844. doi: 10.1001/jama.2023.1044, 36735264 PMC10015303

[ref14] Sun Y. S. H. Hong J. Zhou Y. (2022). Hypergraph-based representation learning for multi-omics data integration in precision medicine. Brief. Bioinform. 23:bbac315.35945147

[ref15] Wei J. Wang X. Schuurmans D. Bosma M. Xia F. Chi E. . (2022). Chain-of-thought prompting elicits reasoning in large language models. Adv. Neural Inf. Process. Syst. 35, 24824–24837. doi: 10.52202/068431-1800

[ref16] Yang X. Chen A. PourNejatian N. Shin H. C. Smith K. E. Parisien C. . (2022). A large language model for electronic health records. NPJ Digit. Med. 5:194. doi: 10.1038/s41746-022-00742-2, 36572766 PMC9792464

[ref17] Yu X. Wang J. Hong Q.-Q. Teku R. Wang S.-H. Zhang Y.-D. (2022). Transfer learning for medical images analyses: a survey. Neurocomputing 489, 230–254. doi: 10.1016/j.neucom.2021.08.159

